# Association Study of Serine Racemase Gene with Methamphetamine Psychosis

**DOI:** 10.2174/157015911795017092

**Published:** 2011-03

**Authors:** E Yokobayashi, H Ujike, T Kotaka, Y Okahisa, M Takaki, M Kodama, T Inada, N Uchimura, M Yamada, N Iwata, M Iyo, I Sora, N Ozaki, S Kuroda

**Affiliations:** 1 Department of Neuropsychiatry, Okayama University Graduate School of Medicine, Dentistry and Pharmaceutical Sciences, Okayama, Japan; 2 JGIDA (Japanese Genetics Initiative for Drug Abuse), Japan; 3 Seiwa Hospital, Tokyo, Japan; 4 Department of Neuropsychiatry, Kurume University Graduate School of Medicine, Kurume, Japan; 5 Department of Psychogeriatrics, National Institute of Mental Health, National Center of Neurology and Psychiatry, Kodaira, Japan; 6 Department of Psychiatry, Fujita Health University School of Medicine, Houmei, Japan; 7 Department of Psychiatry, Chiba University Graduate School of Medicine, Chiba, Japan; 8 Department of Neuroscience, Division of Psychobiology, Tohoku University Graduate School of Medicine, Sendai, Japan; 9 Department of Psychiatry, Nagoya University Graduate School of Medicine, Nagoya, Japan

**Keywords:** Methamphetamine psychosis, serine racemase, glutamate, NMDA receptors, SNP.

## Abstract

Experimental studies have demonstrated that not only dopaminergic signaling but also glutamatergic/NMDA receptor signaling play indispensable roles in the development of methamphetamine psychosis. Our recent genetic studies provided evidence that genetic variants of glutamate-related genes such as *DTNBP1*, *GLYT1*, and *G72*, which are involved in glutamate release and regulation of co-agonists for NMDA receptors, conferred susceptibility to methamphetamine psychosis. Serine racemase converts l-serine to d-serine, which is an endogenous co-agonist for NMDA receptors. Three single nucleotide polymorphisms (SNPs) in the promoter region of the serine racemase gene (*SRR*), rs224770, rs3760229, and rs408067, were proven to affect the transcription activity of *SRR*. Therefore, we examined these SNPs in 225 patients with methamphetamine psychosis and 291 age- and sex-matched controls. There was no significant association between methamphetamine psychosis and any SNP examined or between the disorder and haplotypes comprising the three SNPs. However, rs408067 was significantly associated with the prognosis for methamphetamine psychosis and multi-substance abuse status. The patients with C-positive genotypes (CC or CG) of rs408067 showed better prognosis of psychosis after therapy and less abuse of multiple substances than the patients with GG genotypes. Because the C allele of rs408067 reduces the expression of *SRR*, a lower d-serine level or reduced NMDA receptor activation may affect the prognosis of methamphetamine psychosis and multiple substance abuse. Our sample size is, however, not large enough to eliminate the possibility of a type I error, our findings must be confirmed by replicate studies with larger samples.

## INTRODUCTION

Methamphetamine is widely abused around the world [1, 2]. In Japan, it has been the most popular illegal abused substance since World War II, and its use produces serious social problems [3, 4]. Development of novel or innovative medicines that are more efficient for treatment of methamphetamine use disorders is necessary. Methamphetamine has a strong pyschostimulant effect on the CNS, and elucidation of the neuronal mechanisms underlying methamphetamine-induced psychological dependence and psychosis is considered a top priority. Many experimental studies using a behavioral sensitization model, an animal model of methamphetamine dependence and psychosis, have revealed that the dopamine system in the brain plays central and indispensable roles in the induction and expression of methamphetamine use disorders [5]. These findings seem reasonable because methamphetamine is an indirect dopamine agonist. In addition, animal studies have also revealed that glutamate and itsN-methyl-D-aspartate (NMDA) receptors play important roles in methamphetamine use disorders. Thus, methamphetamine administration enhanced glutamate release in the rat accumbens and ventral tegmentum area (VTA) [6, 7]. Repeated amphetamine administration results in enhanced neuronal responsiveness to locally applied glutamate in the VTA [8, 9] and frontal cortex [10]. Repeated methamphetamine administration induces behavioral sensitization, which was blocked by co-administration of MK-801 (dizocilpine) or CPP (D, L-3 [(+/-)-2-carboxypiperazin-4-yl]-propyl-1-phosphonic acid), an NMDA-receptor antagonist by systemic administration or microinjection into the VTA [11-14]; further, infusion of iGluR antagonists into the accumbens reduced the motor stimulant effects of amphetamine [15]. Reductions in glutamate transmission by administration of the glutamate-release inhibitor riluzole [16], the glutamate transporter activator MS-153 [17], infusion of an AMPA (α-amino-3-hydroxy-5-methyl-4-isoazole-proprionic acid)/KA (kainite) antagonist into the accumbens [18], and virally mediated overexpression of EAAT2 (a glial high affinity glutamate transporter, SLC1A2) in the accumbens [19] all have been shown to attenuate the development of amphetamine conditioned place preference. Mice deficient in GluRepsilon1, which is a human ortholog of the NR2A subunit of the NMDA receptor, showed attenuation of acute methamphetamine-induced hyperlocomotion and low dose methamphetamine-induced sensitization [20]. These experimental findings indicate that enhanced glutamate transmission *via* NMDA and/or AMPA receptors due to methamphetamine administration is essential for induction of sensitization and conditioning to the drug [5]. Therefore, it is possible that genomic variants of genes encoding molecules involved in glutamate signaling may affect individual susceptibility to methamphetamine dependence and psychosis. Based on this hypothesis, we previously analyzed the genetic associations of several genes related to glutamate signaling, such as *DTNBP1*, *GLYT1*, and *G72*, and found that all these genes were significantly associated with methamphetamine use disorders [21-23]. Serine racemase is an enzyme that converts l-serine to d-serine and increases the level of d-serine, which is a co-agonist for the NMDA receptor and regulates activity of NMDA receptors in the brain [24, 25]. Therefore, we investigated a possible association between the serine racemase gene (SRR) and methamphetamine psychosis.

## MATERIALS AND METHODS

### Subjects

The subjects comprised 225 patients with methamphetamine psychosis (181 male and 44 female; mean age ± SD, 37.5 ± 11.9) and 274 age-, gender-, and geographical-origin matched healthy controls (217 male and 57 female; mean age ± SD, 37.6 ± 13.1). All subjects were unrelated Japanese born and living in relatively restricted areas of Japan. All patients were out- or inpatients in psychiatric hospitals of the Japanese Genetics Initiative for Drug Abuse (JGIDA). Consensus diagnoses of the patients were made by two trained psychiatrists according to ICD-10 criteria on the basis of unstructured interviews and medical records. The patients suffered from F15.5 (methamphetamine psychosis) and also F15.2 (methamphetamine dependence). The controls had no individual or family history of drug dependence or major psychotic disorders such as schizophrenia or bipolar disorder. This study was approved by the ethics committee of each JGIDA institution. After a complete description of the study to the subjects, written informed consent was obtained.

### Clinical Phenotypes

The patients with methamphetamine psychosis were divided into subgroups according to the following clinical characteristics: age at first consumption of methamphetamine (younger or older than 20 years), latency to the onset of psychotic symptoms after the first consumption (less than or more than 3 years), prognosis of methamphetamine psychosis after therapy (transient type: psychotic symptoms disappeared within 1 month after discontinuance of methamphetamine use and treatment with an antipsychotic, or prolonged type: psychotic symptoms lasted more than 1 month even after discontinuance of methamphetamine use and treatment with an antipsychotic), spontaneous relapse to a psychotic state, and the presence or absence of multiple-substance abuse. Detailed information on the clinical subgroups of methamphetamine dependence and psychosis was reported elsewhere [22, 26].

### Genotyping

Peripheral blood was obtained from the subjects, and genomic DNA was extracted from peripheral leukocytes using the standard method. Based on our previous study of schizophrenia, three SNPs in the promoter region, rs224770, rs3760229, and rs408067, were analyzed. The first two SNPs were genotyped using TaqMan^®^-based techniques (Applied Biosystems 7300/7500 Real Time PCR System, Applied Biosystems Japan Ltd., Tokyo, Japan) with TaqMan probes (assay ID C_1027003_10 for rs224770 and C_1625595_10 for rs3760229). The polymerase chain reaction was carried out in a total volume of 7 μL containing 3.2 μL TaqMan^® ^2×Universal PCR Master Mix, 0.32 μLTaqMan^®^ probe, and 20 ng of DNA. The amplification protocol was denaturation at 95°C for 10 min, followed by 40 cycles of denaturation at 92°C for 15 sec, and annealing and extension at 60°C for 1 min. Rs408067 was genotyped by the restriction enzyme fragment length polymorphism method. PCR was carried out in a total volume of 15 ml with 10% dimethyl sulfoxide and 0.75 units of Taq DNA polymerase in the reaction mixture with mismatch probes (forward: 5' CCG GCC GCA CCT CTG CGC ACG CGC GGC 3', reverse: 5' CCC CCT GCC GCC CTC CCT TTC CGA ACG 3'). Initial denaturation was performed for 5 min at 95°C, then 35 cycles of 30 sec of denaturing at 95°C, 30 sec of annealing at 68°C, and 30 sec of extension at 72°C were performed, followed by a final extension at 72°C for 5 min. The PCR products were then analyzed on 4.5% agarose gels after digestion with Ehe*I*.

### Statistical Analysis

Deviation of the genotypes from Hardy-Weinberg equilibrium was tested using a chi-square goodness-of-fit test. The statistical significance of the difference was assessed by a chi-square test (genotype comparison) or log likelihood ratio test (allele comparison) at a significance level of 0.05. The pairwise linkage disequilibrium (LD) and haplotype frequencies were estimated by the EH algorithm using the SNPalyze program (Dynacom Co., Mobara, Chiba, Japan).

## RESULTS

Genotype distributions and allele frequencies of the three SNPs of the *SRR* gene of the patients with methamphetamine psychosis and control subjects are shown in Table **1**. The genotype distributions of the control subjects did not deviate from Hardy-Weinberg equilibrium at any of the three SNPs (rs224770, χ^2^=0.86, P=0.35; rs3760229, χ^2^=0.11, P=0.74; rs408067, χ^2^=0.48, P=0.49). In the single marker analysis, no SNP showed a significant difference between patients and control subjects in the frequency distribution of the genotype (rs224770, χ^2^=1.23, P=0.54; rs3760229, χ^2^=2.51, P=0.29; rs408067, χ^2^=1.21, P=0.55) or allele (rs224770, χ^2^=0.73, P=0.39; rs3760229, χ^2^=0.026, P=0.87; rs408067, χ^2^=0.022, P=0.88).

Table **2** shows the results of the pairwise linkage disequilibrium (LD) among the three SNPs of the *SRR* gene using the D’ and r^2^ values as an index. The D’ and r^2^ between each pair of SNPs were above 0.7 and 0.3, respectively, which indicated strong LD between the SNPs. Accordingly, the three SNPs are located on the same LD block. The LD structure found in the present study is consistent with our previous study of schizophrenia [27]. Then, we analyzed three marker haplotypes consisting of rs224770, rs3760229, and rs408067 (Table **3**). Five haplotypes were found, and the G-T-G haplotype was the major one. There was no significant difference in estimated frequencies of haplotypes between the patients with methamphetamine psychosis and controls (global permutation *p* value = 0.068).

Effects of the SNPs on several clinical phenotypes, age at first abuse of methamphetamine, latency of psychosis from the first abuse, prognosis of psychotic symptoms after therapy, complication of spontaneous relapse of psychosis and multi-substance abuse status, were analyzed. Allelic and genotypic distributions of rs224770 and rs3760229 were not associated with these clinical phenotypes (Table **4A** and **4B**); however, rs408067 was significantly associated with a prognosis for methamphetamine psychosis and multi-substance abuse status (Table **4C**). The patients with C-positive genotypes (CC or CG) of rs408067 showed the transient type of psychosis significantly more frequently after standard therapy with antipsychotics than the patients with the GG genotype (p=0.039). This indicated that C-positive genotypes of rs408067 may have a better response to antipsychotic treatment and better prognosis of methamphetamine psychosis. The patients with the C allele or C-positive genotypes of rs408067 also showed significantly less heavy multi-substance abuse than those with the G allele or GG genotype (p=0.036 and 0.029, respectively). They abuse fewer illegal substances other than methamphetamine, such as morphine, cocaine, or cannabis.

## DISCUSSION

The present study revealed that the SRR gene did not affect susceptibility to methamphetamine use disorders, but it was associated with several clinical phenotypes of methamphetamine dependence and psychosis. Carriers of the C allele of rs408067 showed the less prolonged type of methamphetamine psychosis compared with non-carriers, indicating a better prognosis after therapy with antipsychotics and less multi-substance abuse of illegal drugs other than methamphetamine. Our previous study [27] found that rs408067, which is located in the promoter region of the SRR gene, may be functional because an *in vitro* luciferase assay showed that the C allele had 60% lower promoter activity than the G allele did. Therefore, having the C allele of rs408067 may produce a decrease in the SRR level and d-serine conversion, and a decreased level of d-serine, a co-agonist for NMDA receptors, may result in reduced activity of NMDA receptors. Therefore, reduced NMDA receptor signaling due to possession of the C allele of rs408067 of the SRR gene may be a protective factor against a worse prognosis of methamphetamine psychosis and heavy multi-substance abuse behaviors.

A set of molecules are involved in the regulation of glutamate and NMDA receptor signaling. One of those is dysbindin, which is encoded by the DTNBP1 gene. Dysbindin was shown to be involved in glutamate release from synaptic terminals [28]. Recently, we found that the polymorphisms of P1635 (rs3213207) and SNPA (rs2619538) and the three-locus haplotype of P1655 (rs2619539)-P1635-SNPA of the* DTNBP1* gene showed significant associations with methamphetamine psychosis [22]. The P1635 polymorphism of DTNBP1 was also associated with a worse prognosis for methamphetamine psychosis. We also reported that two genes involved in regulation of d-serine were associated with methamphetamine psychosis [21, 23]. They were the GLYT1 and G72 genes. *GLYT1* encodes glycine transporter type 1, which locates in glia close to NMDA receptors and reuptakes glycine and d-serine; both are allosteric co-agonists of NMDA receptors. *GLYT1* was significantly associated with methamphetamine use disorders. *G72* encodes an activator of d-amino acid oxidase [29], which metabolizes d-serine by oxidation. Two haplotypes of the G72 gene in the 5’ and 3’ regions were significantly associated with methamphetamine psychosis [21]. Thus, our previous and present findings consistently indicate that genetic variants that may affect neural signaling of glutamate and NMDA receptors affect susceptibility to methamphetamine-use disorders including psychosis and associated clinical characteristics.

The sample size in the present study was not too small, but it was not large enough to exclude the possibility of a type I error. Therefore, our findings must be replicated in an independent larger sample and other populations.

## Figures and Tables

**Table 1:** Association between the SRR Gene and Methamphetamine Psychosis

**Rs 2224770**								
Group	N	Genotype			Allele		
		G/G (%)	G/A (%)	A/A (%)	p value	G (%)	A (%)	p value
Control	291	169 (58.1)	101 (34.7)	21 (7.2)		439 (75.4)	143 (24.6)	
METH	221	118 (53.4)	87 (39.4)	16 (7.2)	0.54	323 (73.1)	119 (26.9)	0.39
**Rs 3760229**								
Group	N	Genotype			Allele		
		T/T (%)	T/C (%)	C/C (%)	p value	T (%)	C (%)	p value
Control	292	94 (32.2)	140 (47.9)	58 (19.9)		328 (56.2)	256 (43.8)	
METH	225	66 (29.3)	123 (54.7)	36 (16.0)	0.29	255 (56.7)	195 (43.3)	0.87
**Rs 408067**								
Group	N	Genotype			Allele		
		G/G (%)	G/C (%)	C/C %)	p value	G (%)	C (%)	p value
Control	291	169 (58.1)	102 (35.1)	20 (6.9)		440 (75.6)	142 (24.4)	
METH	225	128 (56.9)	86 (38.2)	11 (4.9)	0.55	342 (76.0)	108 (24.0)	0.88

**Table 2:** Linkage Disequilibrium Among the Three SNPs of the SRR Gene

	rs2224770	rs3760229	rs408067	
rs2224770		0.4142	0.9082	r^2^
rs3760229	1		0.3631	
rs408067	0.953	0.9374		
	D'			

**Table 3:** Association of Haplotypes of the SRR Gene with Methamphetamine Psychosis

Haplotype			Control	Patients
rs2224770	rs3760229	rs408067	N=580	N=440
G	T	G	0.552	0.554
A	C	C	0.237	0.233
G	C	G	0.194	0.167
A	C	G	0.009	0.032
G	T	C	0.007	0.009

Global permutation *p* =0.068.

**4A:** Association of Subgroups with Methamphetamine Psychosis According to Clinical Phenotype

rs2224770		Genotype				Allele		
Subgroup	N	G/G (%)	G/A (%)	A/A (%)	p value	G (%)	A (%)	p value
Age at First Use								
20y<	118	62(52.5)	47(39.8)	9(7.6)		171(72.5)	65(27.5)	
20y>=	100	54(54.0)	39(39.0)	7(7.0)	0.98	147(73.5)	53(26.5)	0.82
Latency of Psychosis								
3y<	101	52(51.5)	40(39.6)	9(8.9)		144(71.3)	58(28.7)	
3y>=	92	53(57.6)	35(38.0)	4(4.3)	0.40	141(76.6)	43(23.4)	0.23
Prognosis of Psychosis								
Transient	112	53(47.3)	50(44.6)	9(8.0)		156(69.6)	68(30.3)	
Prolonged	90	55(61.1)	30(33.3)	5(5.6)	0.14	140(30.4)	40(22.2)	0.066
Spontaneous Relapse								
No	116	68(58.6)	41(35.3)	7(6.0)		177(76.3)	55(23.7)	
Yes	81	50(61.7)	26(32.1)	5(6.2)	0.89	126(77.8)	36(22.2)	0.81
Multi-substance abuse								
None or mild	137	67(50.8)	52(39.4)	13(9.8)		186(70.5)	78(29.5)	
Heavy	82	47(57.3)	32(39.0)	3(3.7)	0.22	126(76.8)	38(23.2)	0.15

**4B:** 

rs3760229		Genotype				Allele		
Subgroup	N	T/T (%)	T/C (%)	C/C (%)	p value	T (%)	C (%)	p value
Age at First Use								
20y<	119	34(28.6)	67(56.3)	18(15.1)		135(56.7)	103(43.2)	
20y>=	103	31(30.1)	54(52.4)	18(17.5)	0.91	116(56.3)	90(43.7)	0.88
Latency of Psychosis								
3y<	102	32(31.4)	53(52.0)	17(16.7)		117(57.4)	87(42.6)	
3y>=	95	28(29.5)	55(57.9)	12(12.6)	0.63	111(58.4)	79(41.6)	0.83
Prognosis of Psychosis								
Transient	114	29(25.4)	68(59.6)	17(14.9)		126(55.3)	102(44.7)	
Prolonged	92	32(34.8)	46(50.0)	14(15.2)	0.31	110(59.8)	74(40.2)	0.36
Spontaneous Relapse								
No	120	30(25.0)	69(57.5)	21(17.5)		129(53.8)	111(46.2)	
Yes	94	31(33.0)	51(54.3)	12(12.8)	0.36	113(60.1)	75(39.9)	0.20
Multi-substance abuse								
None or mild	135	37(27.4)	71(52.6)	27(20.0)		145(53.7)	125(46.3)	
Heavy	83	26(31.1)	49(59.0)	8(9.6)	0.13	101(60.8)	65(39.2)	0.14

**4C:** 

rs408067		Genotype				Allele		
Subgroup	N	G/G (%)	G/C (%)	C/C %)	p value	G (%)	C (%)	p value
Age at First Use								
20y<	119	67(56.3)	45(37.8)	7(5.9)		179(75.2)	59(24.8)	
20y>=	103	59(57.3)	40(38.8)	4(3.9)	0.95	158(76.7)	48(23.3)	0.85
Latency of Psychosis								
3y<	103	58(56.3)	40(38.8)	5(4.9)		156(75.7)	50(24.3)	
3y>=	94	56(59.6)	36(38.3)	2(2.1)	0.57	148(78.7)	40(21.3)	0.48
Prognosis of Psychosis								
Transient	114	58(50.9)	52(45.6)	4(3.5)		168(73.7)	60(26.3)	
Prolonged	92	60(65.2)	27(29.3)	5(5.4)	0.056	147(79.9)	37(20.1)	0.14
Spontaneous Relapse					(0.039)			
No	120	65(54.2)	47(39.2)	8(6.7)		177(73.8)	63(26.2)	
Yes	94	56(59.6)	36(38.3)	2(2.1)	0.27	148(78.7)	40(21.3)	0.23
Multi-substance abuse								
None or mild	135	71(52.6)	54(40.0)	10(7.4)		196(72.6)	74(27.4)	
Heavy	83	53(63.9)	29(34.9)	1(1.2)	0.067	135(81.3)	31(18.7)	0.038
					(0.029)			

P values in parentheses indicate those between GG vs C-positive genotypes (GC and CC).
